# Fortified Foods Are Major Contributors to Apparent Intakes of Vitamin A and Iodine, but Not Iron, in Diets of Women of Reproductive Age in 4 African Countries

**DOI:** 10.1093/jn/nxaa167

**Published:** 2020-06-13

**Authors:** Valerie M Friesen, Mduduzi N N Mbuya, Grant J Aaron, Helena Pachón, Olufemi Adegoke, Ramadhani A Noor, Rina Swart, Archileo Kaaya, Frank T Wieringa, Lynnette M Neufeld

**Affiliations:** 1 Global Alliance for Improved Nutrition, Geneva, Switzerland; 2 UMR204 Nutripass, Institut de Recherche pour le Développement (IRD), IRD/Université de Montpellier/SupAgro, Montpellier, France; 3 Rollins School of Public Health, Emory University, Atlanta, GA, USA; 4 Food Fortification Initiative, Atlanta, GA, USA; 5 Oxford Policy Management, Abuja, Nigeria; 6 Africa Academy for Public Health, Dar es Salaam, Tanzania; 7 Harvard TH Chan School of Public Health, Boston, MA, USA; 8 Department of Dietetics and Nutrition, University of the Western Cape, Cape Town, South Africa; 9 Department of Food Technology and Nutrition, College of Agricultural and Environmental Sciences, Makerere University, Kampala, Uganda

**Keywords:** large-scale food fortification, fortified foods, nutrient intakes, iron, vitamin A, iodine, women of reproductive age

## Abstract

**Background:**

Food fortification is implemented to increase intakes of specific nutrients in the diet, but contributions of fortified foods to nutrient intakes are rarely quantified.

**Objectives:**

We quantified iron, vitamin A, and iodine intakes from fortified staple foods and condiments among women of reproductive age (WRA).

**Methods:**

In subnational (Nigeria, South Africa) and national (Tanzania, Uganda) cross-sectional, clustered household surveys, we assessed fortifiable food consumption. We estimated daily nutrient intakes from fortified foods among WRA by multiplying the daily apparent fortifiable food consumption (by adult male equivalent method) by a fortification content for the food. Two fortification contents were used: measured, based on the median amount quantified from individual food samples collected from households; and potential, based on the targeted amount in national fortification standards. Results for both approaches are reported as percentages of the estimated average requirement (EAR) and recommended nutrient intake (RNI).

**Results:**

Fortified foods made modest contributions to measured iron intakes (0%–13% RNI); potential intakes if standards are met were generally higher (0%–65% RNI). Fortified foods contributed substantially to measured vitamin A and iodine intakes (20%–125% and 88%–253% EAR, respectively); potential intakes were higher (53%–655% and 115%–377% EAR, respectively) and would exceed the tolerable upper intake level among 18%–56% of WRA for vitamin A in Nigeria and 1%–8% of WRA for iodine in Nigeria, Tanzania, and Uganda.

**Conclusions:**

Fortified foods are major contributors to apparent intakes of vitamin A and iodine, but not iron, among WRA. Contributions to vitamin A and iodine are observed despite fortification standards not consistently being met and, if constraints to meeting standards are addressed, there is risk of excessive intakes in some countries. For all programs assessed, nutrient intakes from all dietary sources and fortification standards should be reviewed to inform adjustments where needed to avoid risk of low or excessive intakes.

## Introduction

Food fortification is a cost-effective intervention that aims to increase the content of specific nutrients in a widely consumed food to improve the nutritional quality of the food supply (1, 2). Population-based food fortification programs, such as large-scale fortification of staple foods and/or condiments (hereafter referred to as foods), are implemented to address nutrient deficiencies in a population by shifting the distribution of nutrient intakes toward adequacy (3). Globally, mandatory food fortification is legislated in 128 countries for salt, 83 countries for wheat flour, 16 countries for maize flour, and 25 countries for oil (4).

To assess the achievement of food fortification program objectives, we would ideally measure impact on reducing the prevalence and/or severity of nutrient deficiencies or on functional outcomes at the population-level. However, this is costly and it may take substantial time for measurable impacts to be realized (5). Assessing coverage, consumption, and quality of fortified foods is a critical prior step to provide vital information on the contribution of fortified foods to nutrient intakes and the extent to which they meet the intended proportion of dietary needs among target populations. This information can be used to understand a program's performance and potential for biological impact and inform specific needs for program improvement related to design or delivery. It is for this purpose that the Fortification Assessment Coverage Toolkit (FACT) method was developed (6, 7).

Despite the high number of countries that mandate food fortification programs globally, nutrient intakes from fortified foods among target populations have rarely been quantified. Where such information exists, it is mainly from high-income countries (8–11). In low- and middle-income countries (LMICs), individual-level data that would permit these analyses are limited. Where available, data were collected before implementation of the fortification program to estimate potential nutrient contributions of fortified foods, thereby informing the design (12–14). Alternatively, household-level data on food purchasing patterns are often routinely collected in LMICs from household consumption and expenditure surveys (also known as household income and expenditure surveys, household budget surveys, etc.). These data have been increasingly used to estimate potential nutrient contributions of fortified foods to inform program design or model potential impacts of existing programs (15–18). However, they are limited in that they do not always distinguish between food that is potentially fortifiable and that which is not (19), and, in the absence of data on current fortification content in the food supply, they cannot assess nutrient contributions of fortified foods in ongoing programs.

In this article, we quantified the measured and potential intakes of iron, vitamin A, and iodine from fortified foods among women of reproductive age (WRA) using data from FACT surveys conducted in Nigeria, South Africa, Tanzania, and Uganda. In addition, we demonstrate the use and utility of these indicators to assess the performance of fortification programs and identify potential program improvement needs.

## Methods

### Survey design and setting

In 2015, the Global Alliance for Improved Nutrition (GAIN), CDC, and local implementation partners (Oxford Policy Management, University of the Western Cape, Africa Academy for Public Health, and Makerere University) conducted cross-sectional, 2-stage, clustered household surveys in Nigeria, South Africa, Tanzania, and Uganda. Country selection was based on the following criteria: presence of an ongoing large-scale food fortification program that GAIN had supported; lack of recent data on coverage and consumption of fortified foods; and level of prior and existing donor investments. The surveys were designed to determine household coverage of fortified foods and their contributions to key nutrient intakes among WRA. Detailed sampling schemes and coverage results are reported elsewhere (20–24). Briefly, the surveys were state or provincially representative in Nigeria (Kano and Lagos states) and South Africa (Eastern Cape and Gauteng provinces) and nationally representative (stratified by urban and rural) in Tanzania and Uganda. In the first stage, primary sampling units were selected by probability proportional to size (South Africa, Tanzania, and Uganda) or simple random sampling (Nigeria). In the second stage, households in these primary sampling units were randomly selected. Sample sizes were calculated based on a 95% CI, 50% prevalence rate, precision of 0.05–0.065, and design effect of 2, and were adjusted according to country-specific expected response rates.

### Study population

The target study populations included households and WRA (15–49 y of age in Nigeria, Tanzania, and Uganda; 18–49 y of age in South Africa). In each household, the person most knowledgeable about household food preparation and purchasing (≥15 y of age in Nigeria, Tanzania, and Uganda; ≥18 y of age in South Africa) was invited to complete a household questionnaire. In addition, all WRA in each selected household were invited to complete a women's questionnaire.

### Ethical considerations

Ethical approvals were obtained from an academic or national institutional review board in each country and all procedures were followed in accordance with the ethical standards of the responsible institutions. Consent to participate in the survey was obtained from all respondents (verbally in Nigeria and Uganda; in writing in South Africa and Tanzania). Respondents were informed of the nature of the survey, the length of time expected to complete it, and that participation was voluntary and could be withdrawn during any part of the survey.

### Data collection

We collected household-level data on coverage and consumption of fortifiable foods (defined as industrially processed and not made at home) along with other demographic and socioeconomic information described elsewhere (20). Additional individual-level data were collected from WRA on dietary diversity and pregnancy and lactation status. We also collected a sample of each fortifiable food assessed from the household if available. All survey questionnaires and modules were taken from FACT templates (7) and adapted to the local context according to the scope of the fortification program (i.e., number and type of foods) and other country-specific requirements (e.g., culturally appropriate wording of questions and response options). All survey instruments were translated into the common languages spoken in the survey areas and back-translated into English to ensure content validity. Before implementation, they were pilot-tested to finalize the language, wording, and flow of questions and response options. Trained enumerators collected the data using paper forms (South Africa and Uganda) or mobile devices (Nigeria and Tanzania) in a language well understood by the respondent. Skilled field personnel supervised the data collection and ensured data quality through consistency, range, and allowed value checks during data collection for all surveys and also during data entry for paper-based surveys. Up to 2 attempts were made to survey the selected households.

### Laboratory analyses of food samples

Food samples were shipped to reference laboratories in Germany (BioAnalyt, Potsdam and SGS Institute Fresenius GmBH, Taunusstein) and analyzed to determine the added nutrient content from fortification in each individual sample. If flour fortification standards mandated the inclusion of both iron and vitamin A, only iron was measured owing to budget constraints and we assumed vitamin A to be present in the same proportion as added iron relative to the national standard amounts. Vitamin A content was measured in all edible oil and sugar samples and in maize flour samples in Nigeria with iCheck Chroma 3 (25, 26). Iodine content was measured in all salt samples with iCheck Iodine (27). Iron content was measured in wheat and maize flour samples in Tanzania and Uganda with iCheck Iron (28). Iron content was measured in other flour samples (wheat and maize flours in South Africa and wheat and semolina flours in Nigeria) with atomic emission spectroscopy (DIN EN 15510 based on inductively coupled plasma optical emission spectrometry (ICP-OES) method). Because the analysis methods used are unable to distinguish between added and naturally occurring forms of iron, additional unfortified flour samples were collected in each country (1–4 for wheat flour; 4–8 for maize flour; and 2 for semolina flour) and analyzed as composite samples to determine the mean intrinsic iron content of each flour type by country. The mean intrinsic iron content was then subtracted from the total measured iron in each flour sample to estimate the added iron content from fortification.

### Indicator definitions

#### Fortifiable food consumption

The adult male equivalent (AME) method was applied to estimate the daily amount of fortifiable food consumed among WRA in the surveyed households (29). Each member of the household was assigned an age- and sex-specific AME and the AMEs were summed together to calculate a household AME. We estimated the amount of food consumed daily per woman in grams by dividing the woman's AME by the household AME, then multiplying that value by the daily amount of fortifiable food consumed by the household (based on the reported quantity purchased and the duration it usually lasts in the household). WRA from households that reported not consuming the fortifiable food were assigned 0 for daily amount of fortifiable food consumed. Households with no WRA or those with missing data for reported quantity of fortifiable food purchased and/or the duration it usually lasts in the household were excluded. In households that contained multiple WRA, 1 was randomly selected, and her consumption was calculated. Because we used household-level food consumption data and AME assumptions of intrahousehold distribution of the food to estimate individual-level consumption among WRA, all estimates refer to “apparent” consumption.

#### Nutrient intakes

We estimated the daily nutrient intakes from fortified foods among WRA by multiplying the daily apparent consumption of fortifiable food per woman by a fortification content. For measured intakes, we used the median amount quantified from individual food samples collected from households in each country. For potential intakes, we used the targeted amount in the country's national fortification standard that was in effect at the time of the survey (i.e., the midpoint of the required range). If the standard was set as a minimum required value with no upper limit, we set the target at 50% above the minimum on the basis that industry would have to add at least this amount of overage to consistently achieve the minimum content in all food products.

The resulting daily intakes of each nutrient from all fortified foods were combined and reported as percentages of the estimated average requirement (EAR) and recommended nutrient intake (RNI) for WRA according to age and pregnancy and lactation status. Pregnancy and lactation status were only available for the subset of WRA that completed the women's questionnaire, thus all nonsurveyed WRA (i.e., those listed on the household roster but who did not complete the women's questionnaire) were assumed to be nonpregnant and nonlactating. We took RNI values from the WHO and FAO to more accurately reflect international populations (30). For iron, bioavailability was assumed to be 12% in all countries (31). We derived EAR values from the RNI values by dividing by published conversion factors for vitamin A and iodine (1). For iron, the EAR should not be calculated for WRA owing to the skewed distribution of requirements by menstruating women (1); therefore, only the percentage of RNI is reported. In addition, we estimated the prevalence of WRA with nutrient intakes from fortified foods greater than the tolerable upper intake level (UL) based on values taken from the Food and Nutrition Board of the Institute of Medicine (32).

### Data analyses

Data analyses were carried out using SAS version 9.4 (SAS Institute) and Stata version 15.1 (StataCorp LLC), and figures were produced in RStudio version 3.5.1 (R Foundation for Statistical Computing). Medians with IQRs and/or means with 95% CIs were calculated for the primary indicators (i.e., measured fortification contents, apparent consumption of fortifiable foods, and apparent nutrient intakes as a percentage of requirements). For consumption indicators, outliers, defined as values >3 SDs from the mean, were considered implausible and set to missing. We applied appropriate weighting factors to account for the complex sampling designs in the Nigeria, Tanzania, and Uganda surveys. Results from the South Africa survey were not weighted owing to low response rates.

## Results

**Table 1** gives a summary of the survey response rates, women's ages, and food samples collected. Response rates were high in Nigeria (94% in Kano and 92% in Lagos), Tanzania (99%), and Uganda (86%) and low in South Africa (45% in Eastern Cape and 40% in Gauteng). The latter was due predominately to refusal from community leaders or associations and no eligible respondent being available at the time of the survey. Among the surveyed households, the majority contained ≥1 woman of reproductive age (55%–92%). Additional information on fortification program activities in each country and characteristics of the survey populations (i.e., household size, respondent age, proportion at risk of poverty, poor women's dietary diversity score, and rural residence) are reported elsewhere (20). The number of food samples collected from households varied across foods and locations. Salt was the most widely available food across all locations (collected from 72%–86% of households). Comparatively, wheat and maize flour samples were available in <18% and <32% of households, respectively, across all locations (apart from maize flour in South Africa, which was collected from ∼71% of households).

**TABLE 1:** Summary of survey response rates, women's ages, and food samples collected^1^

	Nigeria, Kano	Nigeria, Lagos	South Africa, Eastern Cape	South Africa, Gauteng	Tanzania	Uganda
Planned households	951	951	800	920	1050	1101
Surveyed households^2^	896 (94)	871 (92)	361 (45)	372 (40)	1036 (99)	949 (86)
Surveyed households with ≥1 woman of reproductive age^3^	783 (87)	678 (78)	198 (55)	221 (59)	957 (92)	719 (76)
Age of selected woman of reproductive age, y	28.1 [15–49]	31.8 [15–49]	30.4 [18–49]	33.1 [18–49]	29.9 [15–49]	30.2 [15–49]
Food samples collected^4^						
Wheat flour	110 (12)	15 (2)	39 (11)	4 (1)	191 (18)	47 (5)
Maize flour	33 (4)	2 (<1)	259 (72)	265 (71)	333 (32)	238 (25)
Semolina flour	23 (3)	233 (27)	—	—	—	—
Edible oil	257 (29)	244 (28)	—	—	725 (70)	278 (29)
Sugar	238 (27)	264 (30)	—	—	—	—
Salt	724 (81)	624 (72)	273 (76)	272 (73)	856 (83)	820 (86)

1 All values are *n, n* (%), or mean [range].

2 Percentage reported out of total planned households.

3 Percentage reported out of total surveyed households.

4 Only foods that were fortifiable (i.e., reported to be industrially processed and not made at home) were collected if available in the household; percentage reported out of total surveyed households.

The measured median amounts of vitamin A in wheat flour and oil in Uganda and iodine in salt in South Africa, Tanzania, and Uganda were within the ranges of the national fortification standards (or above the minimum required where no upper limit was provided) (**Table 2**). For all other foods, the measured median amount of nutrient added was below the minimum required in the national standards.

**TABLE 2:** Measured and potential fortification contents of iron, vitamin A, and iodine in individual food samples collected from households^1^

	Nigeria^2^ (Kano and Lagos)		South Africa^2^ (Gauteng and Eastern Cape)		Tanzania		Uganda	
	Measured	Potential	Measured	Potential	Measured	Potential	Measured	Potential
	Median^3^ [IQR]	Target^4^ (standard)	Median [IQR]	Target (standard)	Median [IQR]	Target (standard)	Median [IQR]	Target (standard)
Iron,^5^ ppm								
Wheat flour	11.7 [7.7–33.6]	61.1 (≥40.7)	18.7 [0.0–35.6]	52.5 (≥35.0)	20.9 [9.0–29.6]	40.0 (30.0–50.0)	18.4 [1.1–31.7]	40.0 (25.0–55.0)
Maize flour	—	—	26.3 [16.6–34.3]	52.5 (≥35.0)	0.0 [0.0–0.0]	15.0 (5.0–25.0)	0.0 [0.0–1.2]	15.0 (10.0–20.0)
Semolina flour	23.4 [17.2–35.3]	61.1 (≥40.7)	—	—	—	—	—	—
Vitamin A, ppm								
Wheat flour	2.6^6^ [1.7–7.4]	13.5 (≥9.0)	1.0^6^ [0.0–1.8]	2.7 (≥1.8)	—	—	1.1^6^ [0.1–2.0]	2.5 (1.0–4.0)
Maize flour	0.0 [0.0–0.4]	13.5 (≥9.0)	1.5^6^ [0.9–2.0]	3.0 (≥2.0)	—	—	0.0^6^ [0.0–0.1]	1.0 (0.5–1.5)
Semolina flour	5.2^6^ [3.8–7.8]	13.5 (≥9.0)	—	—	—	—	—	—
Edible oil	3.5 [0.0–30.0]	9.0 (≥6.0)	—	—	4.6 [2.7–12.3]	22.0 (16.0–28.0)	22.4 [11.9–27.2]	32.5 (20.0–45.0)
Sugar	1.0 [0.0–2.1]	11.3 (≥7.5)	—	—	—	—	—	—
Iodine, ppm								
Salt	28.9 [11.9–76.2]	45.0 (≥30.0)	44.5 [25.9–54.0]	50.0 (40.0–60.0)	34.0 [8.2–39.8]	47.5 (25.0–70.0)	36.9 [32.2–41.3]	55.0 (30.0–80.0)

1 ppm, parts per million.

2 Food samples collected from both states or provinces were grouped together for analyses because fortification content is expected to be similar across the country given that food brands are not produced separately for each state/province.

3 Median added nutrient content of all individual household food samples analyzed.

4 Target added nutrient content was set at the midpoint of the required range as per the national standard that was in effect at the time the survey was implemented or 50% above the minimum required content if the standard was set with no upper limit.

5 Measured iron values were adjusted for intrinsic iron by subtracting the estimated mean intrinsic iron content (from analysis of composite samples of nonfortified flours by type from each country) from the total measured iron content.

6 Estimated using measured added iron as a proxy by assuming added iron and vitamin A were present in equivalent ratios that followed the country's fortification standard.

**Table 3** shows the daily apparent consumption patterns of fortifiable foods among WRA. Fortifiable flour consumption varied greatly by location, with the most widely consumed flour being wheat in Kano and Tanzania; maize in Eastern Cape, Gauteng, and Uganda; and semolina in Lagos. The amount of oil consumed daily was similar across Kano, Lagos, and Tanzania but considerably lower in Uganda. Finally, daily salt consumption was approximately twice as high in Kano, Tanzania, and Uganda as in Lagos, Eastern Cape, and Gauteng.

**TABLE 3:** Daily apparent consumption of fortifiable foods by women of reproductive age based on household assessment with the adult male equivalent method^1^

	*n* ^2^	Median [IQR]	Mean (95% CI)
Wheat flour, g/d			
Nigeria (Kano)	770	193 [99.2–288]	202 (185, 219)
Nigeria (Lagos)	668	0.0 [0.0–0.0]	19.5 (13.0, 26.0)
South Africa (Eastern Cape)	198	0.0 [0.0–0.0]	20.8 (13.6, 28.0)
South Africa (Gauteng)	221	0.0 [0.0–0.0]	1.7 (0.5, 2.9)
Tanzania	909	19.3 [0.0–162]	90.0 (74.7, 105)
Uganda	716	0.0 [0.0–0.0]	12.2 (7.1, 17.3)
Maize flour, g/d			
Nigeria (Kano)	780	0.0 [0.0–0.0]	25.3 (11.1, 39.5)
Nigeria (Lagos)	676	0.0 [0.0–0.0]	2.0 (0.3, 3.7)
South Africa (Eastern Cape)	193	88.7 [49.7–133]	101 (91.1, 111)
South Africa (Gauteng)	219	99.1 [69.4–137]	109 (100, 118)
Tanzania	907	0.0 [0.0–116]	60.8 (47.5, 74.2)
Uganda	712	0.0 [0.0–122]	67.4 (49.3, 85.5)
Semolina flour, g/d			
Nigeria (Kano)	781	0.0 [0.0–0.0]	10.7 (4.6, 16.8)
Nigeria (Lagos)	656	56.2 [23.5–136]	88.5 (78.9, 98.1)
Edible oil, mL/d			
Nigeria (Kano)	764	25.8 [16.6–41.4]	29.6 (27.8, 31.4)
Nigeria (Lagos)	669	24.7 [14.0–36.8]	28.7 (26.3, 31.1)
Tanzania	862	19.6 [10.5–36.3]	26.3 (23.7, 29.0)
Uganda	688	5.4 [3.1–9.7]	7.1 (6.1, 8.0)
Sugar, g/d			
Nigeria (Kano)	738	12.2 [7.5–19.8]	14.4 (13.5, 15.3)
Nigeria (Lagos)	644	6.3 [2.6–13.1]	8.8 (8.2, 9.5)
Salt, g/d			
Nigeria (Kano)	749	8.4 [4.5–14.1]	9.9 (8.8, 11.0)
Nigeria (Lagos)	635	3.6 [2.1–5.5]	4.5 (4.2, 4.9)
South Africa (Eastern Cape)	191	4.2 [2.6–6.3]	4.8 (4.4, 5.2)
South Africa (Gauteng)	215	2.7 [1.6–4.3]	3.6 (3.2, 4.0)
Tanzania	869	7.5 [4.6–11.4]	8.8 (8.1, 9.5)
Uganda	697	8.2 [5.9–11.9]	9.4 (8.9, 9.8)

1 Fortifiable is defined as industrially produced and not made at home. Nigeria, Tanzania, and Uganda results were weighted to correct for unequal probability of selection. South Africa results were not weighted owing to low response rates.

2 *n* excludes observations with missing values for reported quantity of fortifiable food purchased and/or the duration it usually lasts in the household and outliers (values >3 SDs from the mean).

Fortified foods contributed modestly to measured iron intakes (0%–13% RNI across all foods and locations); but potential iron intakes if standards are met are higher (14%–65% RNI) in most countries (except Uganda: 0%) (**Table 4**). For example, in Kano, Nigeria, iron intake from fortified wheat and semolina flours would increase from the measured intake of 13% RNI to a potential intake of 65% RNI if flours were fortified to standard. Fortified foods contributed substantially to vitamin A and iodine intakes (20%–125% and 88%–253% EAR, respectively, across all foods and locations); potential intakes if standards were met are higher (53%–655% and 115%–377% EAR, respectively) and would exceed the UL among 18%–56% of WRA for vitamin A in Nigeria and 1%–8% of WRA for iodine in Nigeria, Tanzania, and Uganda (Table 4).

**TABLE 4:** Apparent iron, vitamin A, and iodine intakes (measured and potential) from fortified foods as a percentage of requirements among WRA^1^

		Nutrient intake as % of EAR, median [IQR]		Nutrient intake as % of RNI, median [IQR]		% of women with nutrient intake > UL	
	*n*	Measured^2^	Potential^3^	Measured	Potential	Measured	Potential
Iron							
Nigeria (Kano)^4^	782	—^5^	—^5^	12.9 [6.3–22.0]	64.8 [31.6–107]	0.0	1.0
Nigeria (Lagos)^4^	677	—^5^	—^5^	7.0 [2.3–14.7]	19.1 [6.3–40.1]	0.0	0.1
South Africa (Eastern Cape)^6^	198	—^5^	—^5^	10.7 [6.6–17.2]	22.2 [13.2–36.8]	0.0	0.0
South Africa (Gauteng)^6^	221	—^5^	—^5^	11.0 [7.6–17.0]	21.9 [15.1–34.0]	0.0	0.0
Tanzania^6^	931	—^5^	—^5^	0.0 [0.0–15.2]	13.5 [0.0–33.8]	0.0	0.0
Uganda^6^	719	—^5^	—^5^	0.0 [0.0–0.0]	0.0 [0.0–11.0]	0.0	0.0
Vitamin A							
Nigeria (Kano)^7^	783	125 [73.0–204]	655 [379–1072]	89.5 [52.1–146]	468 [271–765]	0.1	56.4
Nigeria (Lagos)^7^	678	103 [45.7–205]	297 [138–595]	73.3 [32.6–147]	212 [98.3–425]	0.1	17.7
South Africa (Eastern Cape)^6^	198	39.3 [23.9–56.0]	80.2 [49.0–115]	28.0 [17.1–40.0]	57.3 [35.0–82.4]	0.0	0.0
South Africa (Gauteng)^6^	221	37.0 [26.5–55.8]	74.0 [53.1–112]	26.4 [19.0–39.8]	52.8 [37.9–79.7]	0.0	0.0
Tanzania^8^	862	19.5 [9.8–38.9]	93.1 [46.7–186]	13.9 [7.0–27.8]	66.5 [33.4–133]	0.0	0.0
Uganda^9^	719	26.8 [12.6–50.9]	53.2 [25.3–95.8]	19.2 [9.0–36.3]	38.0 [18.1–68.4]	0.0	0.0
Iodine^10^							
Nigeria (Kano)	749	183 [102–319]	286 [159–496]	131 [72.9–228]	204 [114–355]	0.2	7.7
Nigeria (Lagos)	635	87.8 [51.8–143]	137 [80.6–222]	62.8 [37.0–102]	97.7 [57.6–158]	0.0	0.8
South Africa (Eastern Cape)	191	169 [103–252]	190 [116–283]	120 [73.7–180]	135 [82.8–202]	0.0	0.0
South Africa (Gauteng)	215	102 [65.1–180]	115 [73.1–202]	72.7 [46.4–128]	81.7 [52.2–144]	0.0	0.0
Tanzania	869	213 [133–337]	297 [186–471]	152 [95.1–240]	213 [133–336]	0.1	4.3
Uganda	697	253 [171–356]	377 [255–531]	181 [122–254]	269 [182–379]	0.7	4.3

1 RNI values were taken from the WHO and FAO (30) (for iron, bioavailability was assumed to be 12% in all countries); EAR values were derived from RNI values by dividing by published conversion factors (1). UL values were taken from the Food and Nutrition Board of the Institute of Medicine (32). Nigeria, Tanzania, and Uganda results are weighted to correct for unequal probability of selection. South Africa results are not weighted owing to low response rates. AME, adult male equivalent; EAR, estimated average requirement; RNI, recommended nutrient intake; UL, tolerable upper intake level; WRA, women of reproductive age.

2 Based on daily apparent consumption of the fortifiable (i.e., industrially processed and not made at home) food from the AME method multiplied by the median nutrient content quantified from individual food samples collected from households.

3 Based on daily apparent consumption of the fortifiable (i.e., industrially processed and not made at home) food from the AME method multiplied by the target nutrient content as per the national standard that was in effect at the time of the survey.

4 From wheat and semolina flours.

5 EAR cannot be derived from RNI for WRA owing to the high variability and skewed distribution of requirements for iron (1).

6 From wheat and maize flours.

7 From wheat flour, maize flour, semolina flour, oil, and sugar.

8 From oil.

9 From wheat flour, maize flour, and oil.

10 From salt for all countries.

**FIGURE 1:**
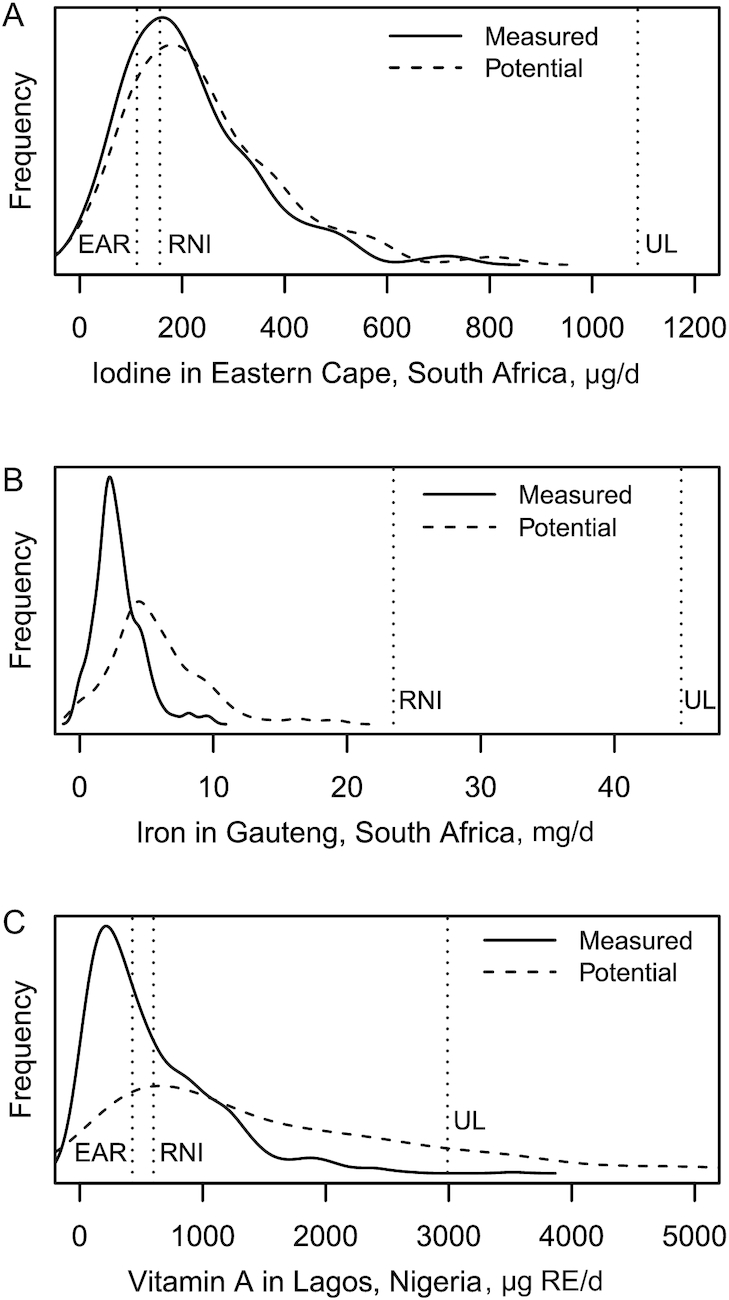
Apparent intakes of iodine from fortified salt in Eastern Cape, South Africa (A); iron from fortified wheat and maize flours in Gauteng, South Africa (B); and vitamin A from fortified oil, sugar, wheat, maize, and semolina flours in Lagos, Nigeria (C). Intakes were estimated by multiplying the apparent amount of fortifiable food consumed daily (based on household assessment using the adult male equivalent method) by a fortification content (measured, based on the median amount quantified from individual food samples collected from households; and potential, based on the targeted amount in national fortification standards). RNI values were taken from the WHO and FAO (30) (for iron, bioavailability was assumed to be 12% in all countries); EAR values were derived from RNI values by dividing by published conversion factors (1). UL values were taken from the Food and Nutrition Board of the Institute of Medicine (32). EAR, estimated average requirement; RE, retinol equivalents; RNI, recommended nutrient intake; UL, tolerable upper intake level.

It is also helpful to visualize the contribution of fortified foods to nutrient intakes as a distribution of intakes because this permits a rapid assessment of the current performance of the programs relative to their design (i.e., potential for impact). As such, the measured and potential intakes from fortified foods are also shown as distributions in relation to the EAR, RNI, and UL among WRA for 3 select examples in **Figure 1** (for all country/nutrient combinations, see **Supplemental Figures 1–15**). In Figure 1A, the measured and potential iodine intakes from fortified salt in Eastern Cape, South Africa are nearly aligned and exceed the EAR and RNI in most of the population without exceeding the UL. In Figure 1B, the potential iron intake from fortified foods in Gauteng, South Africa is greater than the measured intake; however, in both cases, intakes are below the RNI. In Figure 1C, the measured and potential vitamin A intakes from fortified foods in Lagos, Nigeria exceed the EAR and RNI in most of the population and also exceed the UL; however, the potential intake is greater and would result in 18% of the population with intakes above the UL.

## Discussion

In this analysis, we have shown that fortified foods are major contributors to apparent vitamin A and iodine intakes in diets of WRA in the programs assessed, whereas contributions to iron intakes are relatively modest. Our findings in addition suggest that if constraints to achieving the target fortification content as per the national standards are addressed, fortified foods have potential to contribute further to intakes of all nutrients in most countries. However, this increase could result in excessive vitamin A intakes in Nigeria and iodine intakes in Nigeria, Tanzania, and Uganda among some WRA.

Iron fortification programs in all countries are underperforming (contributing 0%–13% RNI), due primarily to poor fortification content combined with low consumption of the fortifiable foods in some locations. Vitamin A fortification programs are similarly underperforming in all countries (except Uganda) and are primarily constrained by suboptimal fortification content, yet are still making substantial contributions to vitamin A intakes across all locations (20%–125% EAR). Finally, iodine fortification programs are performing in accordance with their design (i.e., meeting fortification standards) in nearly all locations (with some room for improvement in Nigeria) and, as a result, are making substantial contributions to iodine intakes (88%–253% EAR).

The implications of these results vary by nutrient and food in each country program, as follows.

### Iron

In most countries with iron fortification programs, the iron content in the fortified foods must be increased to meet standards in order to produce positive impacts on iron intakes. To achieve this, effective and functioning regulatory monitoring systems are necessary to ensure the food industry is compliant with the fortification standards, which may require increasing technical capacity, accountability, and funding and reducing political barriers (33). Alternatively, in Uganda, the current analyses indicate poor selection of foods for fortification given the low amounts of fortifiable wheat and maize flours consumed at population-level. In this case, even if industry is compliant with the fortification standards, the potential contributions to iron intakes will be negligible, as shown. That said, it is likely that the household-level assessment methods used in these analyses underestimated the amount of fortifiable wheat flour consumed in the population because other studies in Uganda reported that bread and other wheat flour–containing products prepared from fortifiable wheat flour are widely purchased (34). However, further research is still needed to confirm the fortification content of the flour in these prepared foods to enable the assessment of their contributions to iron intakes.

### Vitamin A

In Nigeria, the results indicate that the vitamin A fortification standards are likely set too high for the current consumption patterns of the 5 foods mandated for fortification. Currently, this does not pose a major threat to excessive intakes because the food industry is not compliant with the standards. However, if the programs were to improve and become compliant, potential vitamin A intakes would be a major concern because they would result in a large proportion of WRA with intakes above the UL (18% and 56% in Lagos and Kano, respectively) before accounting for other sources of vitamin A in the diet. When fortifying multiple foods with the same nutrient, it is critical to set standards such that the total intake from all fortified foods, as well as other dietary sources and supplements combined, does not consistently exceed the UL in the target population (1). This has been raised as a potential concern in Nigeria and recent efforts have been made to coordinate the array of existing programs including food fortification and supplementation (35), underscoring the need for effective program monitoring and enforcement systems to tackle this critical issue. Vitamin A intakes above the UL may have adverse effects; therefore, in all countries it is recommended to review the vitamin A intake results in the context of all vitamin A sources in the diet and adjust fortification standards as needed to ensure safety over time.

### Iodine

Unlike iron and vitamin A, there are few naturally occurring dietary sources of iodine; therefore, it is appropriate in most countries to design a fortification program such that the sole fortification food (i.e., salt) provides 100% of the EAR. In Nigeria, Tanzania, and Uganda, potential iodine intakes if standards were met would result in a small proportion of WRA with intakes above the UL (1%–8%). Although the risks associated with excessive iodine intakes are not a concern in most people (36), sodium intakes >2 g/d (equivalent to 5 g salt) were attributed to 1.65 million deaths from cardiovascular disease globally in 2010 (37). As a result, many countries, including South Africa, are implementing salt reduction strategies that highlight the need to monitor iodine intakes from fortified salt over time and adjust standards as needed to account for changing consumption patterns (38).

### Strengths and limitations

The main strengths of this analysis were the use of standardized indicators from the FACT method to assess the apparent consumption of fortified foods and their contribution to nutrient intakes, because it allows for comparability across countries over time. The study had several limitations. The survey response rates in both South African provinces were low; therefore, the results may not be reflective of the entire populations. In flour fortification programs where standards mandated the addition of both iron and vitamin A, vitamin A was estimated indirectly using measured added iron as a proxy, which may have overestimated the true vitamin A content because it was not possible to confirm its presence in the premix in those countries and some may have been destroyed during storage. Moreover, further research is needed to confirm the extent to which the estimated vitamin A intakes from fortified oil are likely to reflect true intakes given that it is light-sensitive and predominantly used (and sometimes reused) for cooking rather than being directly consumed, which have been shown to result in significant losses in vitamin A before consumption (39). The AME method likely has precision and estimation errors because consumption of fortified foods varies within households and across food items made with them. For example, it may overestimate the amount consumed of foods that are not equally distributed within the household, and it may underestimate the amount consumed of foods that are commonly purchased outside the household and/or in the form of already prepared products because these were not accounted for in the household questionnaire. Finally, the total nutrient intake from dietary sources other than fortified foods was not collected in the surveys owing to the high-cost technical and financial resources required to collect and analyze them (40). As a result, it was not possible to ascertain the extent to which the additional nutrient intake coming from fortified foods is enough to fill the nutrient gaps in the diet (i.e., shift intakes from inadequate to adequate) or result in additional intakes above the UL.

### Conclusions

Food fortification programs have potential to reduce nutrient deficiencies by improving nutrient intakes in target populations. Our findings demonstrate the extent of the measured and potential apparent nutrient intakes from fortified foods and highlight several programs at risk of low or excessive nutrient intakes owing to poor program design and/or delivery. In all fortification programs assessed, there is a need to review these results in the context of all nutrient sources in the diet, validate them with biochemical data on nutrient status to confirm problem nutrients, and redesign programs to achieve optimal nutrient intakes where necessary. Moving forward, global research priorities for all fortification programs should include the routine assessment of program outcomes, including quality, consumption, and nutrient contribution of fortified foods, through ongoing monitoring efforts. By filling these research gaps, fortification programs will be able to generate the information needed to track progress, identify and overcome barriers, and ultimately achieve their goal of filling the nutrient gaps and improving health outcomes in the population.

## Supplementary Material

nxaa167_Supplemental_Files

## Notes

Supported by a Bill & Melinda Gates Foundation grant.

Author disclosures: The authors report no conflicts of interest.

Supplemental Figures 1–15 are available from the “Supplementary data” link in the online posting of the article and from the same link in the online table of contents at https://academic.oup.com/jn/.

Abbreviations used: AME, adult male equivalent; EAR, estimated average requirement; FACT, Fortification Assessment Coverage Toolkit; GAIN, Global Alliance for Improved Nutrition; LMIC, low- and middle-income country; RNI, recommended nutrient intake; UL, tolerable upper intake level; WRA, women of reproductive age.
