# Supplementary material for: Fortified Foods Are Major Contributors to Apparent Intakes of Vitamin A and Iodine, but Not Iron, in Diets of Women of Reproductive Age in 4 African Countries
Source: J Nutr. 2020 Jun 13;150(8):2183–90. doi: 10.1093/jn/nxaa167 (PMC7398785; doi:10.1093/jn/nxaa167)
Supplement: nxaa167_Supplemental_Files [file nxaa167_supplemental_files.zip › SupplementalFigure12.pdf]

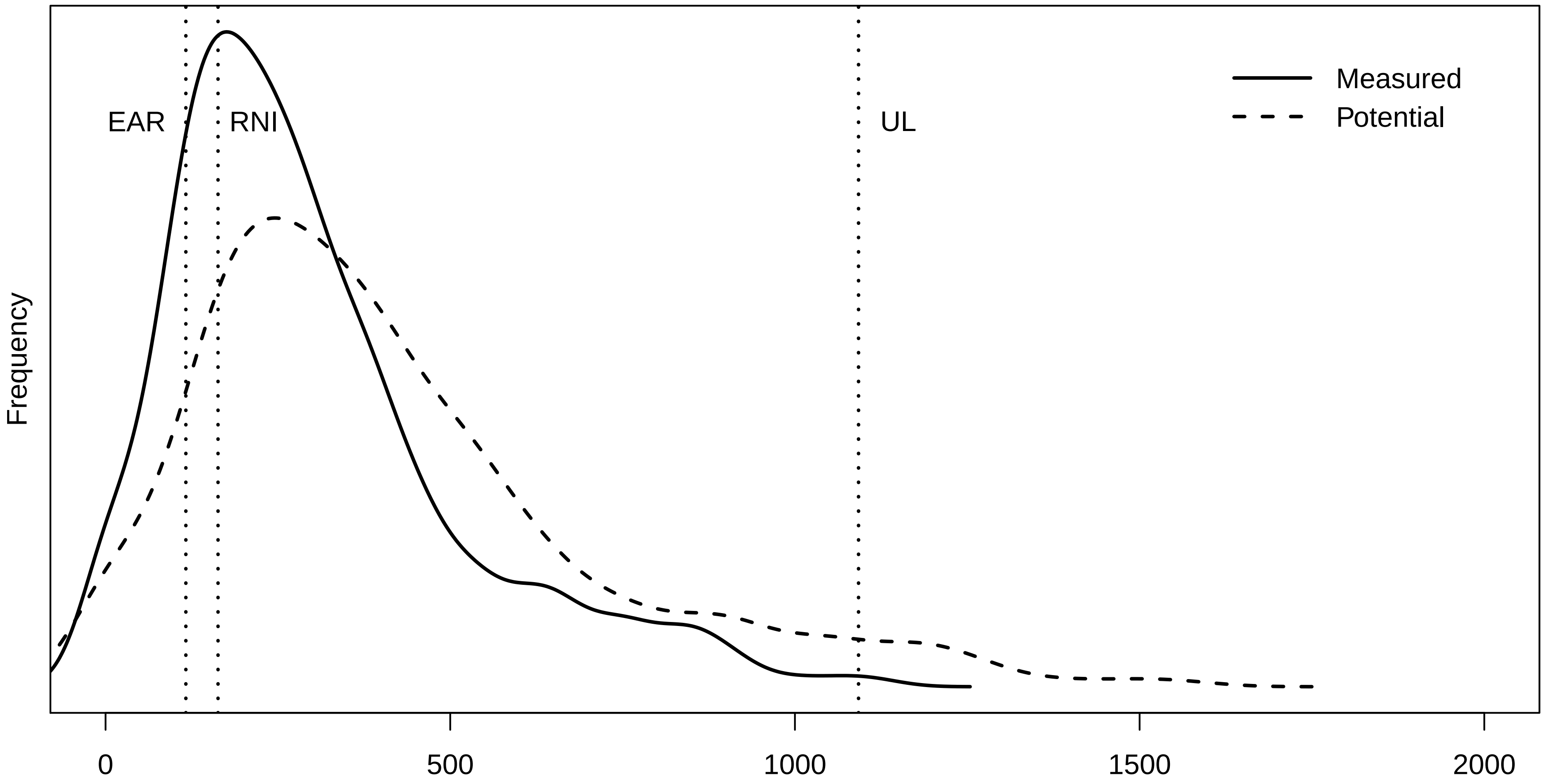

SUPPLEMENTAL FIGURE 12. Apparent iodine intakes from fortified salt among women of reproductive age in Tanzania (µg/day). EAR, estimated average requirement (1); RNI, recommended nutrient intakes (30); UL, tolerable upper intake level (32).
